# Ethical Implications of the Mild Encephalitis Hypothesis of Schizophrenia

**DOI:** 10.3389/fpsyt.2017.00038

**Published:** 2017-03-13

**Authors:** Rita Riedmüller, Sabine Müller

**Affiliations:** ^1^Mind and Brain Research, Department of Psychiatry and Psychotherapy, CCM, Charité – Universitätsmedizin Berlin, Berlin, Germany

**Keywords:** schizophrenia, ethics, medical, mild encephalitis, stigmatization, compulsory treatment, autoimmune encephalitis

## Abstract

Schizophrenia is a serious mental disease with a high mortality rate and severe social consequences. Due to insufficient knowledge about its etiopathogenesis, curative treatments are not available. One of the most promising new research concepts is the mild encephalitis hypothesis of schizophrenia, developed mainly by Karl Bechter and Norbert Müller. According to this hypothesis, a significant subgroup of schizophrenia patients suffer from a mild, but chronic, form of encephalitis with markedly different etiologies ranging from viral infections, traumas to autoimmune diseases. This inflammatory process is thought to occur in the beginning or during the course of the disease. In this article, we investigate the consequences of the mild encephalitis hypothesis of schizophrenia for the scientific community, and evaluate these consequences ethically. The mild encephalitis hypothesis implies that schizophrenia would no longer be considered an incurable psychiatric disorder. Instead, it would be considered a chronic, but treatable, neurological disease. This paradigm shift would doubtlessly have significant consequences: (1) major reforms would be necessary in the theoretical conceptualization of schizophrenia, which would challenge the psychiatric diagnostic systems, Diagnostic and Statistical Manual of Mental Disorders version 5 and ICD-10. (2) Psychotic patients should be treated in interdisciplinary teams, optimally in neuropsychiatric units; additionally, specialists for endocrinology, diabetology, and cardiology should be consulted for the frequently occuring somatic comorbidities. (3) Current diagnostic procedures and (4) therapies would have to be modified significantly. (5) There might be repercussions for the pharmaceutical industry as well: first, because old drugs with expired patent protection could partly replace expensive drugs and, second, because there would be a demand for the development of new anti-inflammatory drugs. (6) Legal evaluation of compulsory treatment orders might have to be reconsidered in light of causal therapies; leading to increased legal approval and reduced need for compulsory treatment orders due to better patient compliance. (7) The social inclusion of patients might improve, if treatment became more effective regarding cognitive and social functioning. (8) The stigmatization of patients and their relatives might decrease.

## Introduction

Schizophrenia is a severe psychiatric disease that affects about 1% of the worldwide population. It is characterized by hallucinations, delusions, disorganization of thought and behavior, depression, flattened affect, cognitive disorders, and social withdrawal. In most cases, the disease takes a chronic, relapsing-remitting course with progressive cognitive decline and a significantly reduced life-expectancy. Most patients are excluded from society because of their bizarre and sometimes frightening behavior, and—depending on the societal system—end up in special care homes, asylums or jails, on the street, or are even executed. Human Rights Watch (1) states that “US prisons and jails have taken on the role of mental health facilities” as a consequence of the “limited availability of community-based outpatients and residential mental health programs and resources.” In the USA, direct and indirect costs of schizophrenia amounted to approximately 62.7 billion in 2002 (2). Between 1.5 and 3% of the total national health-care expenditures are spent on patients with schizophrenia (3).

The pathophysiology of schizophrenia is still unknown (4). Standard therapies against schizophrenia are only symptomatic and provide control rather than cure (5). Antipsychotics, the standard drugs, are criticized because of severe side effects, including metabolic syndrome and brain atrophy (6, 7). More and more evidence supports the hypothesis that schizophrenia is a neurological disease rather than a psychosocial disorder. One important piece of evidence is the recent discovery of anti-NMDA receptor encephalitis (8), which causes psychotic states leading, in some cases, to a misdiagnosis of schizophrenia (9).

One of the most promising new research concepts is the mild encephalitis hypothesis of schizophrenia, developed mainly by the German psychiatrists, Karl Bechter and Norbert Müller (10–15). According to this hypothesis, a significant subgroup of patients with schizophrenia suffer from a mild, but chronic form of encephalitis which can have quite different etiologies ranging from viral infections, traumas to autoimmune diseases. At least in a subgroup of schizophrenia patients, inflammatory processes occur in the beginning or during the course of the disease (16–20). Therefore, anti-inflammatory drugs might be effective. Indeed, several small, but high quality studies have shown significant effectiveness of several anti-inflammatory drugs such as aspirin and *N*-acetylcysteine as add-on medication to antipsychotic drugs, particularly for first-episode psychosis patients (18, 20, 21). Since different etiologies (genetically caused, immunological, growth factor-related, acquired, etc.) can underlie psychotic symptoms, a careful differential diagnosis is necessary. The aim of this article is not to provide a comprehensive review, but to focus on arising ethical questions.

The mild encephalitis hypothesis implies that schizophrenia would no longer be considered an incurable psychiatric disorder, but instead, a chronic, and in many cases, treatable neurological disease. With this paradigm shift, significant consequences could be expected for (1) the theoretical conceptualization of schizophrenia, which will challenge the psychiatric diagnostic systems, Diagnostic and Statistical Manual of Mental Disorders version 5 (DSM-5) and ICD-10; (2) the medical discipline in charge of schizophrenia patients; (3) the diagnostic procedures; (4) the therapies; (5) the pharmaceutical industry; (6) the legal evaluation of compulsory drug treatment; (7) the social inclusion of patients; and (8) the stigmatization of patients and their relatives.

We proceed with a general description of schizophrenia (part 2). Then, we present the mild encephalitis hypothesis of schizophrenia, discussing the available scientific evidence (part 3). Finally, we investigate which consequences could be expected of the mild encephalitis hypothesis of schizophrenia, and evaluate these consequences ethically (part 4).

## Schizophrenia

The recent psychiatric diagnostic systems ICD-10 and DSM-5 ground on a nominalistic concept of mental diseases, which is agnostic with regard to etiology and neuropathology.

Symptoms of schizophrenia are categorized into two classes: positive symptoms describe an excess of normal functions (e.g., delusions, hallucinations, disorganized speech, and behavior) and negative symptoms a decline or loss of normal functioning (diminished emotional expression or avolition).

The DSM-5 defines schizophrenia by six criteria (A–F) (22). Criterion A requires for the diagnosis of schizophrenia that at least two of five characteristic symptoms (1. delusions, 2. hallucinations, 3. disorganized speech, 4. grossly disorganized or catatonic behavior, and 5. negative symptoms) are present for a significant portion of time during a 1-month period (or less if successfully treated). Criterion B refers to social/occupational dysfunction, and Criterion C defines the required duration of symptoms. Criteria D–F distinguish schizophrenia from other disorders. Particularly, Criterion E excludes a diagnosis of schizophrenia if the disturbance is attributable to physiological effects of a substance or another medical condition.

### Clinical Course

Psychotic features of schizophrenia typically appear between the late teens and mid-30s. Sustained recovery occurs in less than 30%; relapse rates are very high and reach approximately 80% (4). In the majority of patients, the illness becomes chronic with severe social consequences: in Europe, only 20% of people with schizophrenia are employed. In the USA, 20% are homeless 1 year after the diagnosis (4). Individuals with schizophrenia are at increased risk to become violent offenders (23). The risk of committing a violent offense is 4.6-fold increased in men, and even 23.2-fold in women (24).

People with schizophrenia have high comorbidity rates for further psychiatric disorders, particularly substance abuse, obsessive-compulsive disorder, and panic disorder (22).

Apart from psychotic symptoms, people with schizophrenia often suffer from inappropriate affect, disturbed sleeping patterns, lack of interest in eating, somatic concerns, impulsiveness, reduced attention, and deficits in Theory of Mind (22).

Furthermore, schizophrenia is associated with general medical risk factors: a higher prevalence of obesity, diabetes mellitus (partly due to atypical antipsychotics), and hypertension. These risk factors lead to an elevated risk for chronic illnesses, such as coronary heart disease, metabolic syndrome, and pulmonary diseases (22).

Patients with schizophrenia have twofold to threefold higher mortality rates compared to the general population. Life expectancy is reduced by 10–25 years (25). Four main reasons contribute to the higher mortality rate: comorbid physical illnesses, insufficient physical health care, adverse effects of antipsychotic medication, and suicides (25). Approximately 20% of patients with schizophrenia attempt suicide, while 5–6% die by suicide (22).

### Genetic and Environmental Factors

The heritability of schizophrenia is about 80%, but the search for its genetic basis has been frustrating (26). Schizophrenia is a polygenetic disorder. A genome-wide association study discovered 108 schizophrenia-associated genetic loci, many of which are involved in important immune functions, particularly in acquired immunity (27). This finding is conceptually in line with the mild encephalitis hypothesis (13).

The vulnerability-stress model has been the prominent explanatory model for schizophrenia during the past decades (15). Neither the genetic code nor the environment is the sole cause for schizophrenia. Rather the effect of an individual’s genotype depends on environmental exposure and, *vice versa*, the effect of environmental exposure on risk depends on an individual’s genotype (13, 26). The incidence of schizophrenia is twofold to fourfold increased in people living in or raised in urban areas, in migrant and minority ethnic groups, in cannabis users, and in people with childhood adversity (26).

### Neurotransmitter Disturbances and Reduced Brain Volume

Disturbances in neurotransmitters and receptors have been postulated for decades in diverse hypotheses of schizophrenia, especially imbalances in dopamine, glutamate, and serotonin systems. It is assumed that hypofunction of dopaminergic projections from mesolimbic to prefrontal structures causes negative symptoms and that a subcortical excess of dopamine is responsible for positive symptoms (28). The main source of serotonin, the dorsal raphe nucleus, is hypothesized to be chronically upregulated due to stress in schizophrenic patients; this can influence glutamatergic transmission and inhibit dopaminergic neurons, thus causing negative symptoms (29).

Magnetic resonance imaging (MRI) studies demonstrated a progressive loss of brain volume in patients with schizophrenia. Both gray and white matter damage is already present in prodromal and first-episode psychosis patients (6, 7). The reduction of gray matter is associated with elevated peripheral inflammatory markers (7). However, findings of MRI studies are valid on a group level, and do not allow individual diagnoses.

### Treatment

*First-generation antipsychotic agents (FGA, typical antipsychotics)*, such as haloperidol, fluphenazine, and chlorpromazine, exert their effects by blocking dopamine receptors and thus decreasing mainly positive symptoms (30). However, FGAs have severe side effects, e.g., deterioration of negative symptoms and cognition, prolactin elevation, acute and chronic movement disorders, such as tremor, rigidity, and tardive dyskinesia (30).

*Second-generation antipsychotic agents (SGA, atypical antipsychotics)*, e.g., clozapine, olanzapine, and quetiapine also block dopamine receptors, but additionally influence serotonin and norepinephrine receptors, which makes them more effective against negative symptoms (28, 30). While SGA do not evoke the typical FGA side effects, they have other severe adverse effects such as agranulocytosis (reduction of white blood cells), weight gain, and alterations in glucose and lipid metabolism (30).

Although brain volume of schizophrenic patients is already reduced before the beginning of antipsychotic medication, both FGAs and SGAs seem to increase this effect (6, 7). The cumulative antipsychotic medication can cause neurocognitive decline, negative and positive symptoms, and worsen psychosocial functioning (6). Cognitive deficits and negative symptoms respond only modestly to antipsychotic medication (4). Neither FGAs nor SGAs improve functional recovery (e.g., employment) (4).

Anti-epileptic agents can be added for reducing aggression and impulsiveness, and antidepressants to reduce depression, anxiety, and if necessary craving for drugs.

Psycho-educational and coping-oriented interventions, cognitive behavioral therapy, cognitive remediation, social skills training, and assertive community treatment can help patients to reintegrate and participate in the community (30). Supportive therapies for family members and patients can enhance medication adherence and help to cope with persistent psychotic symptoms (30).

## The Mild Encephalitis Hypothesis of Schizophrenia

The hypothesis that infections could play a part in the development of schizophrenia is not new: the association between bacterial infections and psychosis was already proposed in 1896 (31). Later on, psychosis and schizophrenic symptoms were hypothesized as consequences of the influenza pandemia in 1918. Unfortunately, these theories were not further investigated due to a lack of relevant treatment methods and the growing prominence of Freudian theories (31). Today, the role of inflammation in psychiatric disorders has become one of the most promising research fields (21).

The mild encephalitis hypothesis published by Karl Bechter in 2001 and updated in the following years, explains the pathophysiology of a subgroup of severe psychiatric disorders, especially of schizophrenic and affective psychoses, in terms of a mild encephalitis. This hypothesis is based on findings from immunology, cerebrospinal fluid (CSF) investigations, imaging studies, and clinical observations. Mild encephalitis is a non-lethal, low grade cellular-infiltrative and/or humoral brain inflammation, possibly accompanied by neurological soft but not hard signs (12). The demarcation between “classical” encephalitis and “mild” encephalitis is important, since “mild” points to the so-called “low-level neuroinflammation” (12). This term is used in clinical publications to describe molecular or cellular abnormalities of minor degree (12).

According to the mild encephalitis hypothesis, the reduced brain volume of schizophrenia patients could be a consequence of mild inflammatory states, which are caused by trauma or various types of toxicity (12). Indeed, elevated cytokine levels are correlated with brain volume loss (7, 12, 14). Inflammation can also disturb brain development of unborn children: during the second half of pregnancy, maternal levels of serum IL-8 (sensitive inflammatory marker) are associated with decreased cortical volumes and an elevated risk for schizophrenia in the offspring (14).

A multitude of factors can trigger mild inflammation, e.g., infections, autoimmunity, toxicity, and trauma; this is modulated by genetic and environmental factors, and immune status (12).

Several lines of evidence support the mild encephalitis hypothesis of schizophrenia.

1. Patients with schizophrenia have increased levels of certain inflammatory markers.
2. Inflammatory processes in the brain can disturb neurotransmitter metabolism.
3. Infections, both prenatal and postnatal, can increase the risk of schizophrenia.
4. There is a correlation between autoimmune diseases and schizophrenia which could be linked to inflammatory events.

### Inflammatory Processes

According to the vulnerability-stress model of schizophrenia, physical and mental stress can cause psychotic episodes. Inflammation could be the missing link between stress and psychosis (15). Stress deteriorates the body’s ability to fight infections, triggers autoimmune activity (32), and increases the production of pro-inflammatory cytokines (15, 16, 31, 33). Pro-inflammatory cytokines are key regulators of inflammation, whereas anti-inflammatory cytokines can inhibit the production of their pro-inflammatory counterparts. Cytokines can affect neurotransmitter levels and microglial activation (33). Microglial cells fight invading antigens, influence growth and apoptosis of neural cells, and can produce cytokines (19). Microglia can be “primed” so that they respond even to a small, second stimulus (14, 15). Thus, cytokine production by microglia can become chronic and also proceed in the absence of the initial trigger.

Schizophrenic patients seem to be in such a heightened inflammatory state: in non-medicated schizophrenic patients, cytokine levels are increased (15, 19). Activated microglia have been detected in patients with recent-onset schizophrenia (15).

### Infections

Maternal immune activation is an important risk factor for schizophrenia and autism in the offspring (15, 34). Inflammation during pregnancy could alter normal neurodevelopment, gene expression, and immune function in the unborn child (34–36). Epidemiological studies, prospective birth studies, and animal studies support the hypothesis that maternal immune activation can cause life-long neuropathology and altered behavior in the offspring. Most maternal infections act as a disease primer (“first hit”) making the individual more susceptible to the effects of genetic mutations and environmental exposures (20, 34).

CNS infections in childhood and in adulthood also elevate the risk of schizophrenia (20, 31). Likely, in both prenatal and postnatal infections, the schizophrenia risk is rather elevated by the immune response (inflammatory cytokines, antibodies), instead of a specific pathogen being responsible for the disease (15, 31, 34–36). As a matter of fact, the risk of developing schizophrenia is associated with the number of severe infections, following a dose–response relationship (31).

Nevertheless, infections with the parasite *Toxoplasma gondii* play a special part in schizophrenia. According to a recent meta-analysis, the evidence for an association between schizophrenia and *T. gondii* is “overwhelming” (37): the prevalence of *T. gondii* antibodies is 1.43-fold higher than in controls (37). A similar association exists for obsessive–compulsive disorder, bipolar disorder, and possibly addiction (37). Presumably, a latent infection with *T. gondii* is reactivated in patients with schizophrenia. The underlying mechanism might be *T. gondii* increases the concentration of dopamine in the brain (38). *Toxoplasma*-infected schizophrenia patients have more severe delusions and a reduced gray matter density in certain parts of the brain compared to *Toxoplasma*-free patients (38).

### Autoimmunity

People with several autoimmune diseases have an elevated risk of developing schizophrenia, and *vice versa*. There are correlations between schizophrenia and many autoimmune diseases, e.g., multiple sclerosis, type 1 diabetes, celiac disease, autoimmune thyroiditis, autoimmune hepatitis, systemic lupus erythematosus, Crohn’s disease, psoriasis, and Guillan–Barré syndrome (32). Multiple sclerosis and schizophrenia might even have similar pathogenetic mechanisms (15). Moreover, multiple sclerosis can at times predominantly present itself with psychiatric features (13).

Linking factors between schizophrenia and autoimmune diseases might be inflammatory events and their consequences (increased permeability of the blood–brain barrier and the intestinal wall, brain-reactive antibodies, increased levels of inflammatory cytokines, and primed microglia) (32). Another explanation might be a genetic vulnerability for dysfunctions of the immune system (32). The correlations between autoimmune disorders and schizophrenia fit well with the mild encephalitis hypothesis, which supposes autoimmunity as a possible trigger for mild inflammatory processes (12).

### Autoimmune Encephalitis

Schizophrenia shares commonalities with autoimmune encephalitis, first described in 2008 (8). In autoimmune encephalitis, antibodies attack neural brain structures (9, 39). For example, anti-NMDA receptor encephalitis is caused by immunoreactivity against a specific part of the NMDA receptor (9, 39). The disease primarily affects females in early adulthood and is accompanied by a tumor in approximately 50% of cases, in this patient group (39). Healthy controls were also found to carry NMDA receptor antibodies, with increasing prevalence depending on age, making the presence of antibodies insufficient for the diagnosis of anti-NMDA receptor encephalitis (40).

Nowadays, additional types of autoimmune encephalitis have been uncovered targeting different neurotransmitter receptors, channel complex associated proteins or other cell structures (39). A growing number of neural antibodies can be detected, due to improved laboratory methods (13).

In anti-NMDAR encephalitis, psychiatric features such as psychosis, confusion, and aggressive behavior are often predominant in the initial phase; hence, patients are initially treated in psychiatric facilities (9). However, as the disease progresses, neurological symptoms, such as tongue thrusting, cheek biting, sucking of lips, hyperkinesia, rigidity, involuntary, stereotyped movements, and spasms, increase. Late stages of the disease are characterized by decreased consciousness and dysregulation of the autonomic center with hyperthermia, elevated heart rate, and reduced breathing (39). Patients can often be treated successfully with immunosuppressive agents such as steroids, intravenous immunoglobulins, and plasmapheresis. Second-line therapy includes pharmacological agents used in cancer treatment and autoimmune disease (9, 39).

Endres et al. (41) found CSF and autoantibody abnormalities in 54.4% of 180 psychotic patients. Bechter (13) found that pathological measures (immunoglobulines, elevated cell counts, inflammatory cytokines, and blood-barrier dysfunctions) in CSF of 41% of schizophrenic and affective spectrum disorder patients, with lower level CSF abnormalities detected in 79% of severe, treatment-resistant cases. Several further studies investigated the prevalence of autoantibodies targeting neural structures in schizophrenia patients, psychiatric patients in general and controls, whereby the results are complex and difficult to interpret (40, 42–45). Presumably, the loss of blood–brain barrier integrity contributes to NMDAR antibody pathologies (40, 43). Antibody-associated mechanisms may be a transient phenomenon in schizophrenia (9), and the concurrent presence of autoantibodies is suggestive of a mild form of encephalitis syndrome (44). Antibody positivity may express itself as a continuum, ranging from relatively “pure” psychotic presentations to catatonia and potentially moribund encephalitis (44).

Just recently, in Germany, the death of a polar bear (ursus maritimus) of the Berlin Zoological Garden, received nation- and worldwide attention: the polar bear, called “Knut,” drowned in 2011 due to seizures and was diagnosed with anti-NMDAR encephalitis post-mortem (46). Knut is the first non-human case of anti-NMDAR encephalitis. It received extensive media coverage and made autoimmune encephalitis known to the wider public.

### Anti-inflammatory Drugs

The mild encephalitis hypothesis is reinforced by clinical studies finding therapeutic benefits when anti-inflammatory agents were added to the antipsychotic medication of schizophrenic patients (18, 20, 21). A meta-analysis of 26 randomized, placebo-controlled double-blind studies describes significant effects for aspirin, estrogens, and *N*-acetylcysteine (NAC, cough syrup) with low to moderate effect sizes (18). Estrogens seem to be effective only in female patients (16, 18); presumably, its effects are hormonal. No statistically significant effects are found for minocycline (antibiotic agent), and omega-3 poly-unsaturated fatty acids (omega-3 PUFAs) (18, 20, 21). However, these substances were shown to be effective in subgroups of patients, particularly in first-episode psychosis patients. Results for celecoxib [a selective cyclooxygenase-2 (COX-2) inhibitor] show a significant advantage in the same subgroup of patients (20, 47). Due to promising but inconclusive effects, further research on these and other anti-inflammatory drugs is necessary.

In a small pilot trial, Tocilizumab, an IL-6 receptor monoclonal antibody, was added to antipsychotics, showing positive effects on cognition without clinically significant side effects [(48), http://ClinicalTrials.gov identifier NCT01696929].

A recent Cochrane study reviewed the effectiveness of antiglucocorticoid substances including 11 studies with 509 patients with psychotic disorders and found some positive effects for mifepristone, although the current data is insufficient to give clear recommendation (49).

A review on nutritional interventions summarizes clinical trials with adjunctive substances, such as antioxidants, vitamin B supplements, neuroprotective, and anti-inflammatory nutrients (alpha-lipoic-acid, melatonin, NAC, vitamin C and E, PUFAs, l-Theanine), as well as exclusion diets (casein-free, glutein-free diet). Based on the reviewed findings, the authors recommend personalized food supplementation, because this strategy could help detect and treat the nutritional deficiencies and food intolerances often encountered in patients. Furthermore, nutritional supplementation could ameliorate symptoms of schizophrenia in some patients (50).

Generally, the effect strength of anti-inflammatory drugs is shown to be greater in first-episode psychosis patients (18). This supports the assumption that inflammatory processes play an important part mainly in the early phase of mild encephalitis schizophrenia.

However, most of these drugs not only exert anti-inflammatory effects, but have further effects that might additionally or alternatively explain their efficacy in schizophrenia: (1) influences on the transmission of dopamine (estrogens, NAC), glutamate (NAC) or serotonin (omega-3-PUFAs), (2) effects on the gut microbiota (minocycline), (3) influence on the body’s stress system (aspirin), (4) neuroprotection (omega-3-PUFAs), (5) enhanced neurogenesis (omega-3-PUFAs), and (6) influence on the composition of cell membranes (omega-3-PUFAs) (16, 18, 21, 51).

### Inflammatory Status in Other Psychiatric Disorders

Inflammatory events could also play an important part in the development of bipolar disorder, major depression, and obsessive–compulsive disorder (21, 33, 52–55). Furthermore, neuroinflammation potentially contributes to neurodegenerative diseases, such as Alzheimer’s disease, Parkinson’s disease, amyotrophic lateral sclerosis, and frontotemporal dementia (56). Some forms of autoimmune encephalitis can even mimic Alzheimer’s disease (57, 58).

Patients with mood disorders suffer more frequently from autoimmune disorders, e.g., multiple sclerosis and diabetes (3-fold higher prevalence), rheumatoid arthritis, systemic lupus erythematosus, and inflammatory bowel disease (33). For example, bipolar disorder is accompanied by several systemic chronic diseases, such as artherosclerosis, hypertension, diabetes, and obesity, which are triggered by inflammatory processes (53). Anti-inflammatory agents (COX-2 inhibitor, acetyl-salicylic acid, fatty acids, and minocycline) are therapeutically effective in patients with bipolar disorder and major depression (33).

### Schizophrenia as a Systemic Disease

According to the mild encephalitis hypothesis, schizophrenia is a systemic disease with preferential involvement of the brain rather than an exclusive brain disease (12, 20, 53). The link between pathologies both in the brain and in the residual body could be the CSF. CSF is produced by the choroid plexus, fills the ventricles and the area around the spinal cord, flows along cranial and spinal nerves, and comes into contact with muscular, subcutaneous, and peripheral neural tissue (12). In 41% of schizophrenic and affective spectrum disorder patients, CSF showed pathological signs (immunoglobulines, elevated cell counts, inflammatory cytokines, and blood-barrier dysfunctions), and 79% of severe, treatment-resistant cases had CSF abnormalities of low level degree (13). Inflammatory messengers likely spread *via* the peripheral cerebrospinal outflow pathway from the CNS to peripheral body compartments. This mechanism could also explain sensory hallucinations experienced by many patients (12). In a study of 180 psychotic patients, 54.4% displayed CSF and autoantibody abnormalities (41).

The understanding of schizophrenia as a systemic disease is further upheld by research on the gut microbiome: inflammatory bowel diseases, such as ulcerative colitis, Crohn’s disease, and irritable bowel syndrome, have a more than 10-fold higher incidence in schizophrenia patients (3.4%) compared to controls (0.3%) (59).

Furthermore, the microbiomes of the oropharynx, pharynx, and intestinal organs differ between schizophrenia patients and controls (59, 60). By profiling oropharyngeal microbiomes with metagenomic sequencing, patients with schizophrenia can be distinguished from controls (60). Hence, a biomarker based on gut microbiota is conceivable (59, 60), and research in this area might facilitate the development of a laboratory test for schizophrenia.

## Ethical Issues of the Mild Encephalitis Hypothesis

If the mild encephalitis hypothesis was further strengthened by clinical evidence, major consequences would have to be expected for (1) the theoretical conceptualization of schizophrenia, (2) the appropriate medical discipline for schizophrenia, (3) the diagnostic procedures, (4) the treatment, (5) the pharmaceutic industry, (6) compulsory treatment, (7) the patients’ social inclusion, and (8) the stigmatization of patients and their relatives.

In the following, we analyze the expected consequences ethically.

### Theoretical Conceptualization of Schizophrenia

The diagnostic term “schizophrenia” can be compared to the umbrella term “bellyache,” for didactic purposes. Rather than delineating certain organs, functional units, and mechanisms that cause the characteristic symptoms, its definition is based solely on symptoms, regardless of their possible causes (4). In an analogous way, the umbrella term “bellyache” describes pain in the abdomen, regardless of its anatomical position, e.g., gastrointestinal tract, Fallopian tube, or the liver, and regardless whether it is caused by infection, autoimmune processes, or poisoning.

Since schizophrenia is not a disease entity, but an umbrella term for different pathologies with common symptoms, subgroups of schizophrenia are feasible; e.g., “schizophrenia should be deconstructed” (61). One subgroup may be caused by mild encephalitis.

For a diagnosis of schizophrenia, DSM-5 requires that the disturbance is not attributable to “another medical condition” (criterion F). Defining “bellyache” analogously, this term could not be used as soon as the pain was attributable to a disorder of the stomach listed in DSM or ICD. The DSM-definition of schizophrenia makes it nearly impossible to explain schizophrenia by reducing the disease to a biological mechanism, since any mechanism would be considered “another medical condition.” This would automatically exclude the diagnostic term: “schizophrenia.” For example, if a patient is diagnosed with mild encephalitis (or, in fact, any other organic pathology), a diagnosis of schizophrenia can no longer be applied (44). Although mild encephalitis is not yet defined as a disease in the ICD-10, it would supposedly be considered a “medical condition” as soon as it was acknowledged that it can cause symptoms of schizophrenia. From that point on, the diagnosis “schizophrenia” could no longer be applied to patients with mild encephalitis.

The psychiatric classification systems DSM-5 and ICD-10 have often been criticized as “descriptive taxonomy based on expressed feelings and observed behavior” (62), as being agnostic on the etiopathogenesis of disorders (63), since its diagnostic tools are insufficiently based on a biomedical understanding of mental illness ((64)). The etiology of psychiatric disorders cannot be elucidated by psychopathology itself (13). The nominalistic approach of the DSM also poses an obstacle for research, slowing the progress of psychiatric science. For example, one reason for the lack of reliable biological tests for psychiatric disorders is the dependence of research criteria on the often too superficial DSM criteria (63).

The Research Domain Criteria (RDoC) project of the National Institute of Mental Health is being developed as an alternative classification system to the DSM-5 system, especially for researchers. The aim of this project is to classify mental disorders based on dimensions of observable behavior and neurobiological measures, e.g., genes, molecules, cells, circuits, physiology, behavior, and self-reports (63, 65). The RDoC could set the foundation for a classification system in which descriptive taxonomy is supported by a biomedical understanding of mental illness. This would further reduce the concerns that psychiatry is merely a tool for social control (64). The main elements of the mild encephalitis hypothesis of schizophrenia could be easily integrated into appropriate RDoC sections, particularly the sections “molecules,” “cells,” and “physiology.”

### The Appropriate Medical Discipline for Treating Schizophrenia

The question of the medical discipline in charge of psychotic patients has far-reaching consequences for the diagnosis, treatment and life-long health care of patients. If patients do not present hard neurological signs such as epilepsy or movement disorders, they are normally hospitalized in psychiatry and diagnosed according to DSM-5 or ICD-10. Many psychiatrists do not routinely perform full physical examinations, since they are less aware of somatic causes of mental illness. Somatic illness is usually addressed as comorbidity, instead of being seen as a symptom of schizophrenia. Only if patients present hard neurological signs, they are referred to neurology, where they undergo CSF analysis, EEG, anti-neural antibody titer analysis, and brain imaging.

This kind of differentiation can become precarious, e.g., for patients with anti-NMDAR encephalitis. Initially, and sometimes throughout the whole course of disease, they may exclusively present psychiatric symptoms and are consequently hospitalized in psychiatric hospitals (9). Since blood tests and CSF analysis for anti-neural antibodies are not standard diagnostic tools in most psychiatric clinics, these patients are at risk of being diagnosed with schizophrenia. As a result of ineffective treatment, they might suffer severe, permanent brain damage or die. Indeed, several cases of patients with anti-NMDAR encephalitis and misdiagnosed with schizophrenia have been reported (42). Somatic examination and adequate antibody screening should become standard procedure in first-episode psychosis patients in order to find possible organic causes (9, 52, 54, 55, 66).

We recommend treating psychotic patients primarily in interdisciplinary teams, optimally in neuropsychiatric units. Psychiatric expertise is necessary for adequately dealing with severe behavioral symptoms and for psychotherapeutic treatment. Neurological expertise is necessary for CSF analysis, imaging data, and further neurological examination in order to adequately identify treatable organic causes (9, 13). Knowledge from both disciplines is needed for optimal, personally tailored pharmacotherapy. Causal therapies might target, e.g., teratomes, parasites, infectious agents, or autoimmune processes. Treating mental illness continuously over longer periods of time is especially challenging since symptoms, such as denial of illness, paranoia, irrational thoughts, deficits in executive function, and disruptive behavior, are often complicating factors (4). Therefore, it is of great importance that patients in early stages of the disease swiftly receive interdisciplinary diagnostics followed by appropriate, possibly causal, treatment.

If more evidence in favor of a mild encephalitis component in schizophrenia was gathered, the diagnostic procedure for patients with psychotic outbreaks would have to change significantly. Three different developments are possible: first, the responsibility for patients with schizophrenia would shift from psychiatry to neurology as it has happened with dementia. Second, the mild encephalitis hypothesis of schizophrenia would contribute to a reunion of psychiatry and neurology. Third, it would support interdisciplinary treatment concepts for schizophrenia.

In addition to psychiatrists and neurologists, internists, and when necessary experts for endocrinology, diabetology, and cardiology should be consulted for somatic comorbidities of schizophrenia, e.g., hypertension, obesity, diabetes mellitus, nicotine dependence, and dyslipidemia. This is of particular importance since physical illnesses are mainly responsible for the twofold to threefold increased mortality rate (25).

To reduce these high mortality rates of patients with schizophrenia and to adequately address their special medical condition, an integrated service provision is required (67). The coordination of mental and physical treatment could be managed by care coordinators (25, 67). Compared to standard care, patients in comprehensive community care settings showed better clinical and functional outcomes (68).

### Diagnostic Procedure

As traditional classification systems such as DSM or ICD will not undergo radical change in the near future, biomedical tests should be added to the existing diagnostic schemes. A first example is the biomarker for schizophrenia based on the gut microbiota (59, 60). Progress in genomics, medical imaging, molecular biology, and cognitive sciences could aid in the development of reliable tests to accurately diagnose psychiatric disorders and to predict treatment response to specific drugs (4, 20, 62, 64). Several diagnostic procedures are recommended based on the mild encephalitis hypothesis.

The International Encephalitis Consortium recommends methods such as the investigation of CSF and serum, MRI, EEG and neurologic examination for diagnosing acute encephalitis (69). However, for mild encephalitis, a standard diagnostic procedure does not yet exist, because relevant changes in disease indicators are small and unspecific, making it difficult to set cut-offs and to detect pathologies (12). Nevertheless, standard diagnostic procedures for acute encephalitis could be adopted for mild encephalitis.

For detecting acute encephalitis, it is recommended to test paired CSF-serum samples for routine parameters, infectious agents, autoantibodies associated with autoimmune encephalitis, and immunoglobulins (69). Similarly, the gold standard for diagnosing schizophrenia of the mild encephalitis type is the investigation of CSF, since it allows the detection of even minor pathological abnormalities (12, 13).

#### CSF and Serum Investigation

Cerebrospinal fluid investigation is the most precise method for detecting inflammation in the central nervous system (13). Although it is not recommended in most guidelines, there are strong arguments for a systematic CSF screening of psychotic patients, especially prior to initiating psychopharmacological treatment (13, 41). With the help of CSF analysis, most neurological disorders can be excluded (13). However, lumbar puncture is not without risks. The most frequent complication is headache (36.5–60%). Rare complications are brain herniation, cardiorespiratory compromise, local or referred pain, hemorrhage, subarachnoid epidermoid cyst, and CSF leak. Serious adverse events caused by infectious agents (e.g., meningitis) occur in <1% (70). When comparing the medical risks and the financial cost with the benefits of routine lumbar puncture in psychotic patients, the benefits overweigh, especially since CSF analysis offers the possibility for an effective, causal treatment.

#### Autoimmune Encephalitis

Each patient with psychosis should be tested for autoimmune encephalitis *via* routine screening for antibodies and inflammatory parameters in serum and CSF, particularly in a first-episode psychotic outbreak. This is necessary to avoid misdiagnosis and consequent inappropriate treatment possibly resulting in long-term disability or even death (41, 66). Patients with pathogenic antibodies can be detected only by screening all first-episode psychosis patients for antibodies (45). With the help of improved laboratory methods to measure antibodies, an increasing number of neural offenders will become detectable (13).

Red flags in the psychopathological status clinically pointing to autoimmune encephalitis, are movement disorders, disturbed consciousness, hyponatriemia, a rapid disease progression, catatonic symptoms, comorbid autoimmune diseases (Hashimoto thyroiditis), focal neurological deficits, MRI-, CSF- and EEG abnormalities, and a very acute disease onset (13). Since not all relevant autoantibodies are known yet, autoimmune encephalitis may be present even if tests for all known autoantibodies are negative. In this case, brain biopsy might confirm autoimmune encephalitis (52).

Disease-specific antibodies for schizophrenia have neither been found in serum nor in CSF (39). In a minority (8%) of schizophrenia patients, NMDAR antibodies are detectable, although they differ from those required for a diagnosis of anti-NMDAR encephalitis (44). These autoantibodies were found in patients with a first episode of psychosis, but not in chronic patients (44). Most likely, autoantibody-associated mechanisms are a transient phenomenon in schizophrenia (9). The presence of autoantibodies in some patients with schizophrenia suggests that these patients have a mild form of encephalitis (44). Whether an individual develops only psychotic symptoms or the full encephalitic syndrome may depend on several factors such as antibody subtype, antibody titer, brain area affected or blood-brain barrier integrity (40, 44).

#### Brain Imaging

In acute encephalitis, MRI can assist to detect abnormalities, demyelination or necrotic lesions, helping to illuminate the pathogenesis (39, 69). However, in mild encephalitis, MRI is not sensitive enough to reliably detect minor lesions and inflammation (12). Nevertheless, signs of mild atrophy, minor local intensities or local swelling could indicate states of mild inflammation (12). Fluorodeoxyglucose positron emission tomography is an important screening tool for yet undetected, but underlying tumors such as teratomas or lymphomas, which can produce antibodies causing psychosis (39). Furthermore, with the advanced dynamic contrast-enhanced MRI, blood-brain barrier disruptions can be investigated (40). Due to good availability and low side effects, neuroimaging is an appropriate method for excluding major brain pathologies (13).

### Treatment

Since the pathophysiology of schizophrenia is still unknown, curative treatment or preemptive interventions are missing (4). Current treatments provide control rather than cure (5). The mild encephalitis hypothesis could change the treatment of schizophrenic patients considerably.

Reducing inflammation is the most important therapeutic consequence of the mild encephalitis hypothesis. It is the prerequisite for controlling both mental symptoms and the comorbidity “metabolic syndrome,” which itself is also associated with mild and chronic inflammation (17, 20). Several treatment strategies are under investigation.

#### Food Supplements

Fishoil (omega-3 PUFAs) might be a preventive drug for patients with a high risk for developing schizophrenia. In a randomized, double-blind, placebo-controlled trial with high risk individuals aged 13–25, intervention with omega-3 PUFAs reduced the risk of progression to psychosis as well as psychiatric morbidity (follow-up 6.7 years). Only about 10% (4/41) in the omega-3 PUFA group transitioned to psychosis, compared to 40% (16/40) in the placebo group (5). Additionally, omega-3 PUFAs reduced positive and negative symptoms, and improved functioning compared to placebo (5, 51). The number needed to treat was 4, which is comparable to atypical antipsychotics (51). The effectiveness of omega-3 PUFAs has also been confirmed for (major) depression by a large meta-analysis (71). Omega-3 PUFAs are key components of brain tissue and, therefore, essential for neural development and function. Presumably, they influence membrane fluidity, receptor responses and modulate dopamine, noradrenaline, and serotonin levels (51). Furthermore, they have anti-inflammatory and anti-apoptotic potential (5). Possible side effects of omega-3 PUFAs, concerning the gastrointestinal tract, are only mild. The advantages of omega-3 PUFAs are their excellent tolerability, public acceptance, relatively low costs, and benefits for general health (21, 51).

Additionally, food supplementation with Vitamine C and *Ginkgo biloba* showed significant effects compared to placebo (20, 72).

#### Anti-inflammatory Medication

Anti-inflammatory medication seems to effectively target the underlying inflammatory states present in a subgroup of patients with schizophrenia (12, 17, 18, 20, 21, 47). Add-on of this treatment regimen was found to be most effective in first-episode psychosis and influenced by the initial inflammatory status of the patient. Therefore, anti-inflammatory medication could be a cause-targeted therapeutic strategy in early phases of the disease to stop its progression (16, 21).

Nevertheless, undesirable side effects have to be considered: aspirin can cause gastrointestinal bleeding; a complication to be avoided by adding gastric protection (16, 18). All in all, the benefit–risk ratio for aspirin is in favor of the prescription (21). NAC (cough syrup), has negligible side effects and offers specific benefits: it can be administered during pregnancy, and might reduce substance abuse, a frequent comorbidity in patients (22). This makes NAC ideally suited as the first-line anti-inflammatory agent against schizophrenia (16, 18).

Celecoxib has rare but severe cardiovascular and gastrointestinal side effects, and should therefore be administered only in acute episodes rather than as long-term medication (21). Minocycline, though positively evaluated in animal and laboratory studies, cannot be recommended as first-line add-on agent because of its unclear efficacy and its significant risks (18, 20, 21).

At the moment, it is difficult to draw strong conclusions about the efficacy and safety of anti-inflammatory agents (16). Thus, no recommendations can be made in general (13). From an ethical point of view, NAC and aspirin can be recommended because of significant effectiveness and good tolerability; omega-3 PUFAs can be recommended because of a good benefit-risk ratio. Two patient groups might especially benefit from add-on of anti-inflammatory medication: schizophrenic patients with predominant immune alterations, and second, first-episode psychosis patients (16, 61). These two patient collectives should be included in future studies as a first step toward personalized medicine for schizophrenia (16, 20, 61).

Ongoing clinical studies include, e.g., studies on aspirin (http://ClinicalTrials.gov identifier: NCT02685748; NCT02047539), Siltuximab (IL-6 monoclonal antibody, NCT02796859), Tocilizumab (IL-6 receptor monoclonal antibody, NCT02874573), and l-tetrahydropalmatine (dopamine antagonist, NCT02118610), *Withania somnifera* (immunomodulator and anti-inflammatory agent, NCT01793935). Trials adding substances to conventional therapies are under current investigation, with promising results, e.g., statines, metotrexate (immunosuppressive and anti-inflammatory agent), glucocorticoids, ibuprofen, and salsalate (non-steroidal anti-inflammatory drug) (20).

#### Antipsychotics

Apart from their evident anti-dopaminergic characteristics, antipsychotics might be effective in schizophrenia due to their anti-inflammatory properties (16, 17, 33). However, many patients refuse antipsychotics due to side effects, particularly in the long run (28, 51, 64). Since the benefit-risk ratio of antipsychotics is unsatisfactory, they should be administered for the shortest time and the lowest dose necessary to avoid severe side effects (6).

#### CSF Filtration

Cerebrospinal fluid filtration could be an add-on therapy in severe therapy-resistant schizophrenic and affective spectrum psychoses with immunological genesis (11). The risks of CSF filtration are justifiable in light of the reduced quality of life and high suicidal risk of psychotic patients (11).

### Consequences for the Pharmaceutic Industry

Current pharmacological treatment options for schizophrenia (mainly antipsychotics) are merely symptomatic, not curative, with limited effectiveness and tolerability. They cannot improve functional recovery, and relapse rates are still about 80% (4). Therefore, better drugs are urgently needed. In agreement with the mild encephalitis hypothesis, drug development focusing on suppressing inflammatory processes might finally open the door to curative treatment.

The main challenge in developing an appropriate anti-inflammatory agent is the agent’s ability to pass the blood–brain barrier.

Current available agents known to cross the blood-brain barrier include: antipsychotics, celecoxib, estrogens, omega-3-PUFAs, minocycline and NAC (18). Aspirin, monoclonal antibodies, and corticosteroids are less able to reach the CNS (18). Despite varying treatment response, older, existing anti-inflammatory drugs with expired patent protection (e.g., NAC, aspirin, celecoxib) could partly replace the more expensive antipsychotic drugs. As there is little incentive for research on old drugs with expired patent protection or cheap food supplements (e.g., omega-3 PUFAs, fishoil), further drug development will likely have to be state-funded.

However, as elaborated above, established anti-inflammatory drugs have a varying efficacy in schizophrenia, and entirely novel, more effective and well tolerable drugs are urgently needed. The demand for new, anti-inflammatory drugs would have significant impact for the pharmaceutical industry. The necessary research would be much more expensive than research on existing drugs. Therefore, depending on the economic and legal conditions of different countries, this research should be conducted by universities, and, if necessary, in combination with the pharmaceutical industry.

### Compulsory Treatment

In response to the UN Convention on the Rights of Persons with Disabilities (73), many countries have modified their laws in order to protect psychiatric patients from being treated compulsorily. For example, the German Federal Constitutional Court acknowledged the “freedom to be ill” in several court rulings on forensic patients, diagnosed with schizophrenia, resisting compulsory treatment with antipsychotics. Besides these individual decisions, the Court decided that the federal laws allowing compulsory drug treatment were unconstitutional. German state parliaments were urged to reformulate their civil commitment laws and implement stricter legal conditions for compulsory treatment. In particular, compulsory treatment was limited to patients incapable of consent; justified by the argument that the freedom to be ill must not be considered detached from the real capacities of free decision-making which may be limited by illness (74).

Although legislation in most Western countries increasingly gives priority to patient autonomy, the concept of autonomy is insufficiently elaborated on. Criteria for the legal concept of “free will” require further explanation. Particularly, input from neurobiology, psychiatry and philosophy is needed. It is important to note that certain psychiatric diagnoses do not exclude freedom of will. Tebartz-van-Elst (75) showed the extent to which free will depends on certain mental functions and those that can be compromised by brain diseases.

We are convinced that individual court rulings would have come to a different conclusion in light of the mild encephalitis hypothesis of schizophrenia, assuming successful treatment of schizophrenia with anti-inflammatory drugs in a relevant subgroup of patients.

First, the Court extensively cited the adverse side effects of antipsychotic drugs. In contrast, current anti-inflammatory drugs such as aspirin and NAC are considered harmless; thus, making a ruling in favor of compulsory treatment more plausible.

Second, the Court’s decision was likely influenced by the fact that antipsychotics are merely a symptomatic, rather than a curative treatment for schizophrenia.

Third, the Court argued with the potential of antipsychotic medication to change the personality. Although it remained inconclusive with regard to the question whether schizophrenia is a psychosocial disorder or a genetically determined condition, as the disease was considered deeply ingrained to an individual’s personality. If the Court adopted the understanding of schizophrenia as an acquired neurological condition, caused or triggered by viruses, parasites, tumors or autoimmune processes, it would not condemn curative drugs. Rather, these drugs would have to be considered *personality-restoring* drugs. Particularly, the involvement of the parasitic protozoan *T. gondii* in schizophrenia might be a convincing argument for the judges, as its survival strategy can be explained by the manipulation hypothesis (38): *T. gondii* is transmitted from intermediate hosts such as mice and rats to its definitive hosts, namely cats, by predation. Hence, *Toxoplasma* relies on cats to eat infected rodents. For facilitating the transmission from the intermediate to the definite host, the parasite manipulates the rodents in several ways: reaction times become prolonged, and the rodents specifically lose their fear to cat odor; this peculiarity is called the fatal attraction phenomenon. The same mechanism is probable in our next of kin: *Toxoplasma*-infected chimpanzees lose the fear to leopard urine (76). Toxoplasmosis can also cause similar behavioral changes in humans: it increases reaction times, resulting in higher probability of traffic and work accidents; additionally, infected men rated the smell of cat urine as relatively more pleasant (38). The suicide rate of infected mothers is twice that of non-infected mothers (77). According to the manipulation hypothesis, these changes could result from the fact that our distant ancestors were also part of the leopards’ prey. In this context, schizophrenia cannot be seen as belonging to the core of the personality.

We expect that the threshold for allowing compulsory treatment would decrease, if legal theorists and high judges accepted the mild encephalitis hypothesis of schizophrenia and if anti-inflammatory drugs were more effective and had lesser adverse effects compared to antipsychotic medications.

However, we expect that the number of compulsory treatment would be reduced significantly in the long run: if patients made the experience that physicians could effectively help them overcome their suffering in the psychotic phases without experiencing the adverse effects of antipsychotics, many would be more compliant with long-term treatment (if necessary). Furthermore, they might sign psychiatric advance directives (Ulysses contracts) for allowing drug treatment in case of another psychotic episode, even against the psychotic will (78). Finally, the better medical treatments can cure the disease, the lesser compulsory treatments would be necessary at all.

We recommend the following: the will of an acutely psychotic individual most likely differs significantly from his or her free will. In a psychotic state, reality perception is largely disturbed; the affect is changed; anxiety and panic dominate, such that the power of judgment is corrupted. Particularly, thought intrusions corrupt the individual’s free will. The affected person is not autonomous, and therefore lacks the capacity to give informed consent. Consequently, a proxy has to decide—but according to the affected individual’s will: first, according to his or her formerly declared will (ideally in an advance directive), second, to his or her assumed will, and third (in case that the latter two are unknown), in his or her best interest.

As we have argued elsewhere (79), respect for autonomy is also a positive duty. If a person’s capability for autonomy is corrupted by a disorder, respect for the person’s autonomy means primarily to restore her capability for autonomy. If restoration of the capability for autonomy is possible with antipsychotics and/or anti-inflammatory medication, then it is a moral obligation to treat the person with these drugs. Once the capability for autonomy is restored, the patient can decide autonomously about his or her further treatment. However, if the patient has ruled out any of these treatments in an advance directive written in a state of legal competence, then this decision has to be respected, as well.

### Social Inclusion

Until the 1970s, people with severe mental illness such as schizophrenia were treated in psychiatric hospitals in great numbers. Due to their often chronic conditions and missing treatment options, they spent most of their lives in sanitariums or asylums (67). With the deinstitutionalization process, the responsibility of care for people with chronic mental illness shifted from hospital- to community-based health services. However, the chronic and severe course of schizophrenia often leads to mental and medical disability, unemployment, homelessness and even incarceration (4). Throughout Europe, less than 20% of people with schizophrenia are employed, and in the USA, people with severe mental illness are three times more likely to be found in the criminal justice system than in hospitals (4).

If new, more effective treatments were developed on ground of the mild encephalitis hypothesis, many patients with schizophrenia of the mild encephalitis type could shift from being chronically ill and mentally disabled to being temporarily ill and treatable patients. Presumably, early interventions targeting underlying pathologies could prevent a chronic course of disease and cognitive impairment, enabling successful reintegration and participation in the community. However, it remains an open question as to how many patients could actually profit from these new therapeutic strategies.

Economically, employment of patients in remission can reduce indirect health costs, since the patient’s productivity is no longer lost and family members can partly pursue their professions (80). Employment improves the patient’s compliance and reduces hospital re-admission rates, which plays an important role in the patient’s quality of life (80).

### Stigmatization

Psychiatric disorders are severely stigmatized in both lay and professional settings (67, 81). Stigmatization means that people are classified and stereotyped due to a negatively connoted attribute. It is often associated with segregation, loss of social status, discrimination in important contexts, and devaluation in a social hierarchy (82). Stigmatized individuals often develop self-stigmatization and withdraw from society. Stigmatization often includes the families of stigmatized persons (courtesy stigma) (83).

The question, whether biological explanations for psychiatric disorders reduce or increase stigma, has been discussed controversially for several decades.

The pessimistic fraction suspects that biologizing psychiatric disorders, particularly “genetic determinism,” intensifies discrimination and stigmatization, because it increases feelings of fear and unfamiliarity (84). Since it assumes an inborn predisposition for deviant behavior, it strengthens the assumption that the disease is unchangeable, persistent, and hereditable (85).

The optimistic fraction is convinced that biological explanations reduce blame against persons with mental disorders, since it assumes that the main reason for stigmatization is the attribution of responsibility for the onset and/or maintenance of the deviant behavior. If a mental disorder is biologically caused, then the person is not responsible for the onset nor the offset or the resulting behavior of the disorder (85).

Empirical research on stigmatization has shown that biological explanations particularly increase stigmatization of diseases which are associated with perceived dangerousness and unpredictability (81). Furthermore, poor treatment success increases stigmatization. Hence, biological explanations might reduce stigmatization as soon as successful treatment options are available (86).

Schizophrenia is associated with (1) perceived high dangerousness and unpredictability, (2) high psychosocial disability and exclusion, and (3) poor treatment success. However, onset and offset responsibility is low. Indeed, it has been shown that stigmatization of people with schizophrenia increases due to biological explanations (86, 87).

The mild encephalitis hypothesis will probably affect stigmatization of schizophrenia in several ways: it does not support genetic determinism, but instead the concept of genetic vulnerability. Therefore, we expect that it will decrease stigmatization in comparison to mainly genetic explanations, but increase it compared to social explanations of schizophrenia.

With the mild encephalitis hypothesis, we do not expect a change concerning the attribution of onset responsibility. We expect a de-stigmatizing effect insofar as it offers some hope for better treatment strategies. Additionally, the patients’ compliance might improve due to less adverse effects of effective drugs, thus, in the long-term, relapse rates might be reduced and cognitive functioning improved. This could decrease the perceived dangerousness and unpredictability of patients and improve their social inclusion. Furthermore, we expect reduced stigmatization of genetic relatives, if the influence of genes is seen not as a determination, but merely as a vulnerability factor.

Finally, we expect a major de-stigmatizing effect as soon as a multi-disciplinary approach in the treatment of schizophrenia is adopted, integrating psychiatry, neurology, and somatic disciplines.

The story of the popular German polar bear, Knut, might also contribute to destigmatization of schizophrenia because some empathy might be transferred from the bear to people suffering from psychosis.

In summary, we expect the mild encephalitis hypothesis to decrease stigmatization of patients with schizophrenia, provided effective drug therapies are developed based on biological findings. Novel therapies based on anti-inflammatory substances might help not all, but a significant number of patients with schizophrenia of the mild encephalitis type.

## Conclusion

We cannot predict the further scientific development in psychiatry. Rather, we investigated the consequences of the mild encephalitis hypothesis of schizophrenia for the scientific community, and evaluated these consequences ethically. Most of these consequences are favorable from an ethical point of view.

Effective treatments of schizophrenia are urgently needed in order to reduce the burden for the patients, their relatives and society in general. For the development of effective treatment strategies, biological research on the etiology of schizophrenia is paramount. Research on both old and new drugs for treating mild encephalitis should be funded by public authorities. Increasing evidence supports the mild encephalitis hypothesis. Therefore, from both a scientific and an ethical point of view, further research on the role of inflammation in the etiology of schizophrenia and other psychiatric and neurological diseases is essential. Knowledge about the biological underpinnings of psychiatric disorders should be transferred into clinical research and clinical practice. Biological tests, particularly paired serum-CSF analyses, should become standard investigations for all psychotic patients in order to identify the appropriate treatment for the individual patient.

## Author Contributions

RR and SM have both contributed to the article with regard to development of ideas and definition of its contents and structure. RR conducted the literature search and evaluation. Both authors read and approved the final manuscript.

## Conflict of Interest Statement

The authors declare that the research was conducted in the absence of any commercial or financial relationships that could be construed as a potential conflict of interest.

## Abbreviations

COX-2, cyclooxygenase-2; CSF, cerebrospinal fluid; CT, computer tomography; DSM-5, Diagnostic and Statistical Manual of Mental Disorders version 5; FDG-PET, fluorodeoxyglucose positron emission tomography; NAC, *N*-acetylcysteine; NMDA, *N*-methyl-d-aspartate; NMDAR, *N*-methyl-d-aspartate receptor; MRI, magnetic resonance imaging; PUFA, poly-unsaturated fatty acids; RDoC, Research Domain Criteria project.
